# The conserved tyrosine residue 940 plays a key structural role in membrane interaction of *Bordetella* adenylate cyclase toxin

**DOI:** 10.1038/s41598-017-09575-6

**Published:** 2017-08-24

**Authors:** Jiri Masin, Jana Roderova, Adriana Osickova, Petr Novak, Ladislav Bumba, Radovan Fiser, Peter Sebo, Radim Osicka

**Affiliations:** 1 Institute of Microbiology of the CAS, v. v. i., Prague, Czech Republic; 2 Faculty of Science, Charles University, Prague, Czech Republic

## Abstract

The adenylate cyclase toxin-hemolysin (CyaA, ACT or AC-Hly) translocates its adenylate cyclase (AC) enzyme domain into target cells in a step that depends on membrane cholesterol content. We thus examined what role in toxin activities is played by the five putative cholesterol recognition amino acid consensus (CRAC) motifs predicted in CyaA hemolysin moiety. CRAC-disrupting phenylalanine substitutions had no impact on toxin activities and these were not inhibited by free cholesterol, showing that the putative CRAC motifs are not involved in cholesterol binding. However, helix-breaking proline substitutions in these segments uncovered a structural role of the Y632, Y658, Y725 and Y738 residues in AC domain delivery and pore formation by CyaA. Substitutions of Y940 of the fifth motif, conserved in the acylated domains of related RTX toxins, did not impact on fatty-acylation of CyaA by CyaC and the CyaA-Y940F mutant was intact for toxin activities on erythrocytes and myeloid cells. However, the Y940A or Y940P substitutions disrupted the capacity of CyaA to insert into artificial lipid bilayers or target cell membranes. The aromatic ring of tyrosine 940 side chain thus appears to play a key structural role in molecular interactions that initiate CyaA penetration into target membranes.

## Introduction

The 1706 residue-long adenylate cyclase toxin-hemolysin (CyaA, ACT or AC-Hly) plays a key role in virulence of pathogenic *Bordetellae* ^1, 2^. CyaA belongs to the family of repeats-in-toxin (RTX) proteins and consists of an N-terminal enzymatic adenylate cyclase (AC) domain of about 400 residues that is fused by an “AC to Hly linker segment” to a pore-forming RTX hemolysin (Hly) moiety of approximately 1300 residues^3, 4^. The Hly moiety then itself consists of a hydrophobic pore-forming domain, a fatty acyl-modified domain, an RTX calcium-binding domain and a C-terminal secretion signal^2, 5^. Hly mediates cell binding of CyaA and delivers its enzymatic AC domain into the cytosol of host cells, where the AC converts ATP to cAMP^6, 7^. In parallel, the Hly moiety oligomerizes into cation-selective pores that permeabilize cell membrane and allow efflux of potassium from cells^8–11^. Both toxin activities then depend on the posttranslational activation of proCyaA to CyaA by covalent fatty-acylation of the ε-amino groups of lysine residues 860 and 983 by a co-expressed protein acyltransferase, CyaC^12–15^.

CyaA exerts a complex array of cytotoxic and immunosubversive activities on host phagocytes^16–23^ to which the toxin specifically binds through the complement receptor 3 (CR3), known also as the α_M_β_2_ integrin, CD11b/CD18, or Mac-1^24, 25^. The initial interaction of CyaA with N-linked oligosaccharides^26, 27^ is followed by specific recognition of CR3 through a specific segment of its CD11b subunit^24, 25^. With one to two orders of magnitude lower efficacy, the CyaA toxin can penetrate also non-myeloid cells that lack the CR3 receptor, or even naked lipid bilayers^28–31^.

Accumulated evidence suggests that at least two alternative and distinct conformers of CyaA co-exist and operate within the target cell membrane. These CyaA conformers exert two parallel and divergent activities, one accounting for translocation of the AC domain across cellular membrane, and the other resulting in formation of a cation selective membrane pore^32–35^. Translocation of the AC domain depends on membrane potential and proceeds with a very short half-time directly across the cytoplasmic membrane of target cells^36, 37^, without the need for endocytosis^29^. Previously we showed that the translocation intermediate of the AC domain itself participates in formation of a novel type of calcium ion conduit across the membrane of monocytic cells^38^. Calcium entry-dependent recruitment of the CyaA-CR3 complex into lipid rafts then follows, where the cholesterol-rich lipid environment promotes translocation of the AC domain across cell membrane^39^.

The presence of cholesterol in the membrane was, indeed, previously found to enhance the binding and lytic capacity of CyaA on erythrocytes and artificial membranes^30, 40^. Moreover, two other RTX toxins, the *Aggregatibacter actinomycetemcomitans* leukotoxin LtxA and the *Escherichia coli* α-hemolysin HlyA, were previously shown to specifically bind cholesterol^41–43^. These toxins possess in their pore-forming domains the so called cholesterol recognition/interaction amino acid consensus (CRAC) motifs. These consist of the L/V-(X)(1–5)-Y-(X)(1–5)-R/K pattern, where (X)(1–5) represents between one and five residues of any amino acid^44^. Single residue substitutions in this CRAC motifs were then found to markedly impair interaction of diverse proteins with target membranes^45, 46^.

Here we examined the function of five putative CRAC motifs that could be predicted in the sequence of the membrane-interacting Hly moiety of CyaA. We show that these motifs do not function as CRAC motifs and are not involved in the interaction of CyaA with target cell cholesterol. Instead, these segments are playing an important structural role in the initial step of toxin penetration into target membrane, thus determining its capacity to translocate the AC domain into cells and to form the cell-permeabilizing pores.

## Results

### The putative CRAC motifs in the Hly moiety of CyaA do not recognize cholesterol

**Figure 1:**
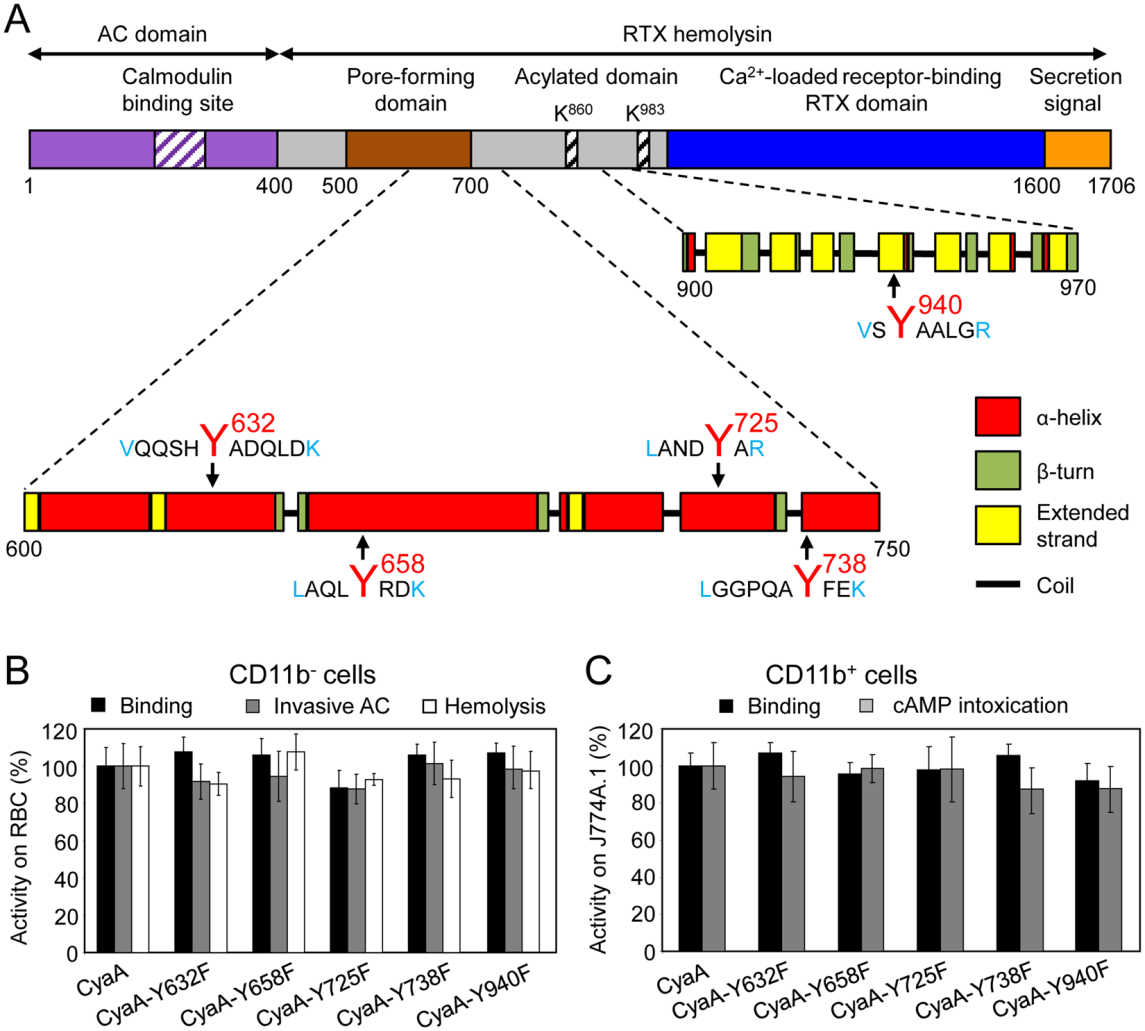
Replacement of the key tyrosine residues by phenylalanine residues in the putative CRAC motifs has no significant effect on the cell binding, AC domain translocation and pore-forming capacities of the CyaA mutants. (**A**) Schematic structure of the CyaA molecule with the predicted CRAC motifs. CyaA is a 1706-residue-long polypeptide that consists of an adenylate cyclase (AC) enzyme domain and a pore-forming RTX hemolysin. The RTX hemolysin moiety contains a hydrophobic pore-forming domain comprising residues 500 to 700, an acylated domain between residues 800 and 1000, where the posttranslational acylation at two lysine residues (K^860^ and K^983^) occurs, a typical calcium-loaded receptor-binding RTX domain and a C-terminal secretion signal. Four putative CRAC motifs were identified in the C-terminal part of the hydrophobic pore-forming domain of CyaA. One putative CRAC motif is located in the C-terminal part of the acylated domain of CyaA. Prediction of secondary structures between residues 600 to 750 and 900 to 970 of CyaA was performed using the SOPMA software^63^. The conserved N-terminal leucine/valine, the central tyrosine (Y^632^, Y^658^, Y^725^, Y^738^ and Y^940^) and the C-terminal lysine/arginine residues of the predicted CRAC motifs are colored. (**B**) Sheep erythrocytes (RBC, 5 × 10^8^/ml) were incubated at 37 °C with 1 µg/ml (5 nM) of intact CyaA or its mutant variants and after 30 minutes, aliquots were taken for determinations of the cell-associated AC activity and of the AC activity internalized into erythrocytes and protected against digestion by externally added trypsin. For determination of hemolytic activity, sheep erythrocytes (5 × 10^8^/ml) were incubated at 37 °C in the presence of 10 µg/ml (50 nM) of intact CyaA or its mutant variants. Hemolytic activity was measured after 4 hours as the amount of released hemoglobin by photometric determination (A_541nm_). (**C**) Binding of intact CyaA or its mutant variants to J774A.1 cells (1 × 10^6^) was determined as the amount of total cell-associated AC enzyme activity upon incubation of cells with 1 µg/ml (5 nM) of the protein for 30 minutes at 4 °C. cAMP intoxication was assessed by determining the intracellular concentration of cAMP generated in cells after 30 minutes of incubation of J774A.1 cells (2 × 10^5^) with four different toxin concentrations from within the linear range of the dose-response curve (100, 50, 25 and 10 ng/ml). (**B**,**C**) Activities are expressed as percentages of intact CyaA activity and represent average values ± standard deviations from at least three independent determinations performed in duplicate with two different toxin preparations.

**Figure 2:**
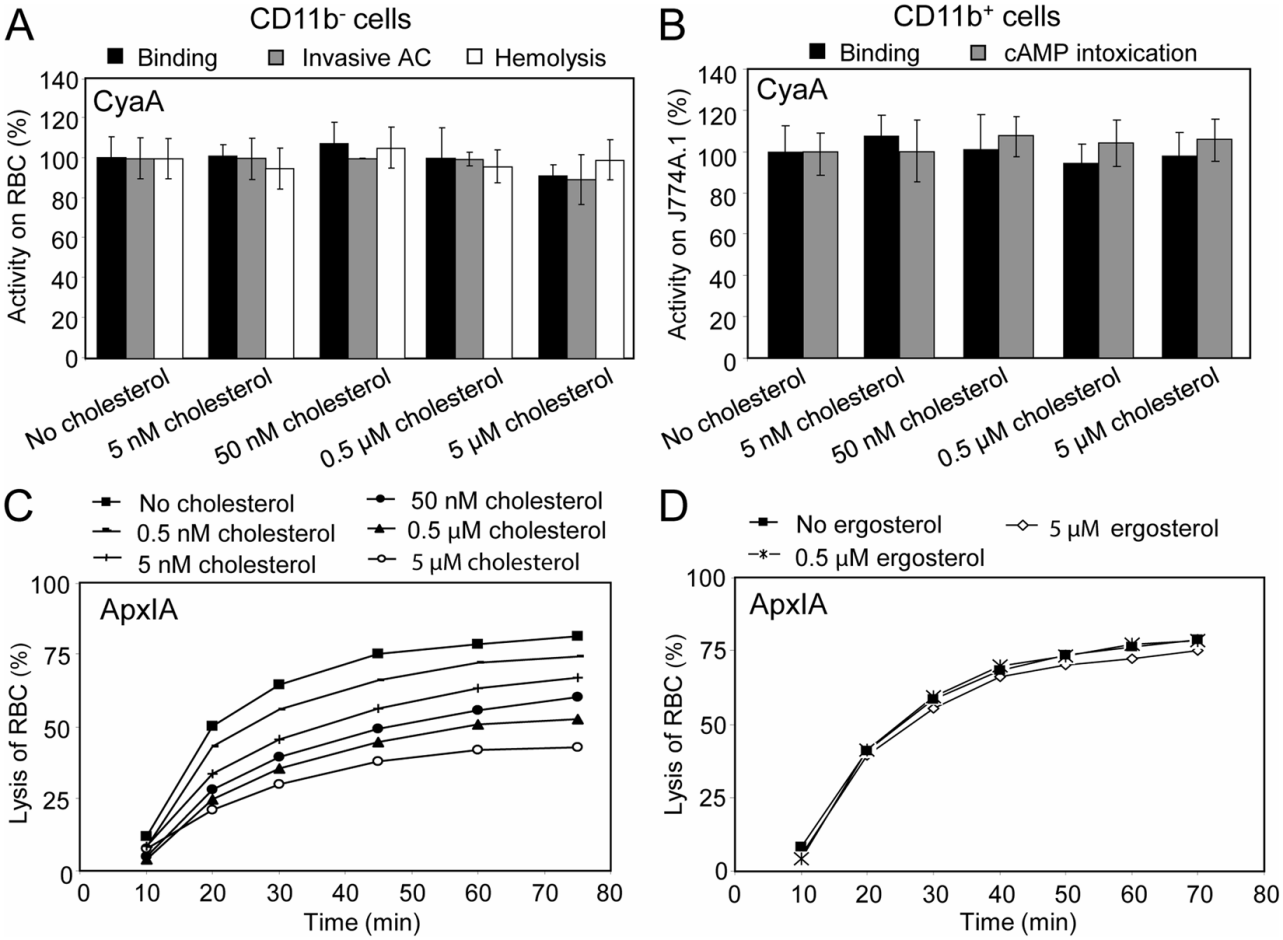
Activity of ApxIA, but not CyaA is inhibited by cholesterol. (**A**) CyaA (5 nM) was preincubated for 20 minutes at room temperature in the presence (5 nM to 5 µM) or absence of cholesterol. Binding and invasive AC activities on sheep erythrocytes were determined as in the legend to Fig. 1. Activities are expressed as percentages of intact CyaA activity in the absence of cholesterol and represent average values ± standard deviations from three independent determinations performed in duplicate. For determination of hemolytic activity, CyaA (50 nM) was preincubated for 20 minutes at room temperature in the presence (5 nM to 5 µM) or absence of cholesterol. Hemolytic activity was measured as in the legend to Fig. 1. (**B**) CyaA (5 nM) was preincubated for 20 minutes on ice in the presence (5 nM to 5 µM) or absence of cholesterol. Binding on J774A.1 macrophages was determined as in the legend to Fig. 1. Before cAMP determination, CyaA (0.5 and 1 nM) was preincubated for 20 minutes at room temperature without or with cholesterol (5 nM to 5 µM). cAMP intoxication was assessed by determining the intracellular concentration of cAMP generated in cells after incubation of J774A.1 cells (2 × 10^5^) with CyaA ± cholesterol for 10 minutes at 37 °C. Activities are expressed as percentages of intact CyaA activity in the absence of cholesterol and represent average values ± standard deviations from three independent determinations performed in duplicate. (**C**,**D**) ApxIA (23 nM) was preincubated for 20 minutes at room temperature with different concentrations of cholesterol (**C**) or ergosterol (**D**). Hemolytic activity was measured as the amount of released hemoglobin in time after addition of ApxIA to 5 × 10^8^/ml of sheep erythrocytes by photometric determination (A_541nm_). Since stock solutions of cholesterol and ergosterol were prepared in 100% ethanol, all CyaA and ApxIA activities in the absence of sterols were measured in the presence of 0.1% ethanol (solvent control).

Analysis of the primary sequence of the membrane-interacting Hly moiety revealed four putative tyrosine residue-containing CRAC motifs in the pore-forming domain and one putative CRAC motif in the acylated domain of CyaA (Fig. 1A). To probe whether these are *bona fide* CRAC motifs and what is their role in toxin activities, we replaced the Y632, Y658, Y725, Y738 and Y940 residues by phenylalanines. Such conservative substitutions were, indeed, reported to disrupt the *bona fide* CRAC motifs^45–47^. To assess the capacity of purified mutant CyaA variants to penetrate target membranes we used as model myeloid phagocytes the mouse J774A.1 macrophages that express the CyaA receptor CR3 (CD11b^+^ cells). In parallel, sheep erythrocytes lacking the CR3 molecule (CD11b^−^ cells) were used as model non-myeloid target cells. Contrary to expectation, none of the Tyr > Phe substitutions in the putative CRAC motifs affected any significantly the cell binding, AC domain translocation or the pore-forming capacities of the CyaA mutants (Fig. 1B,C). Therefore, to corroborate that cholesterol does not play a role in membrane binding and penetration of the Hly moiety of CyaA, the toxin was preincubated with increasing concentrations of free cholesterol for 20 minutes and was then further incubated in the presence of free cholesterol with J774A.1 cells or erythrocytes. As shown in Fig. 2A,B, even 5 µM free cholesterol had no inhibitory impact on membrane insertion of and pore-formation, or AC delivery by CyaA (AC translocation into cytosol of cells and elevation of cAMP concentration). In contrast, as shown in Fig. 2C, exposure of the RTX cytolysin ApxIA of *A. pleuropneumoniae* ^48^ to as little as 0.5 nM free cholesterol reduced its hemolytic activity. This inhibition of ApxIA activity was highly cholesterol-specific, as it was not inhibited by the structurally related ergosterol molecule (Fig. 2D). These data, hence, suggest that the putative CRAC motifs of CyaA do not mediate cholesterol binding and that CyaA insertion into target membranes does not depend on cholesterol binding.

### Tyrosine residues Y632, Y658, Y725 and Y738 are part of α-helical structures involved in AC domain translocation and CyaA pore formation

**Figure 3:**
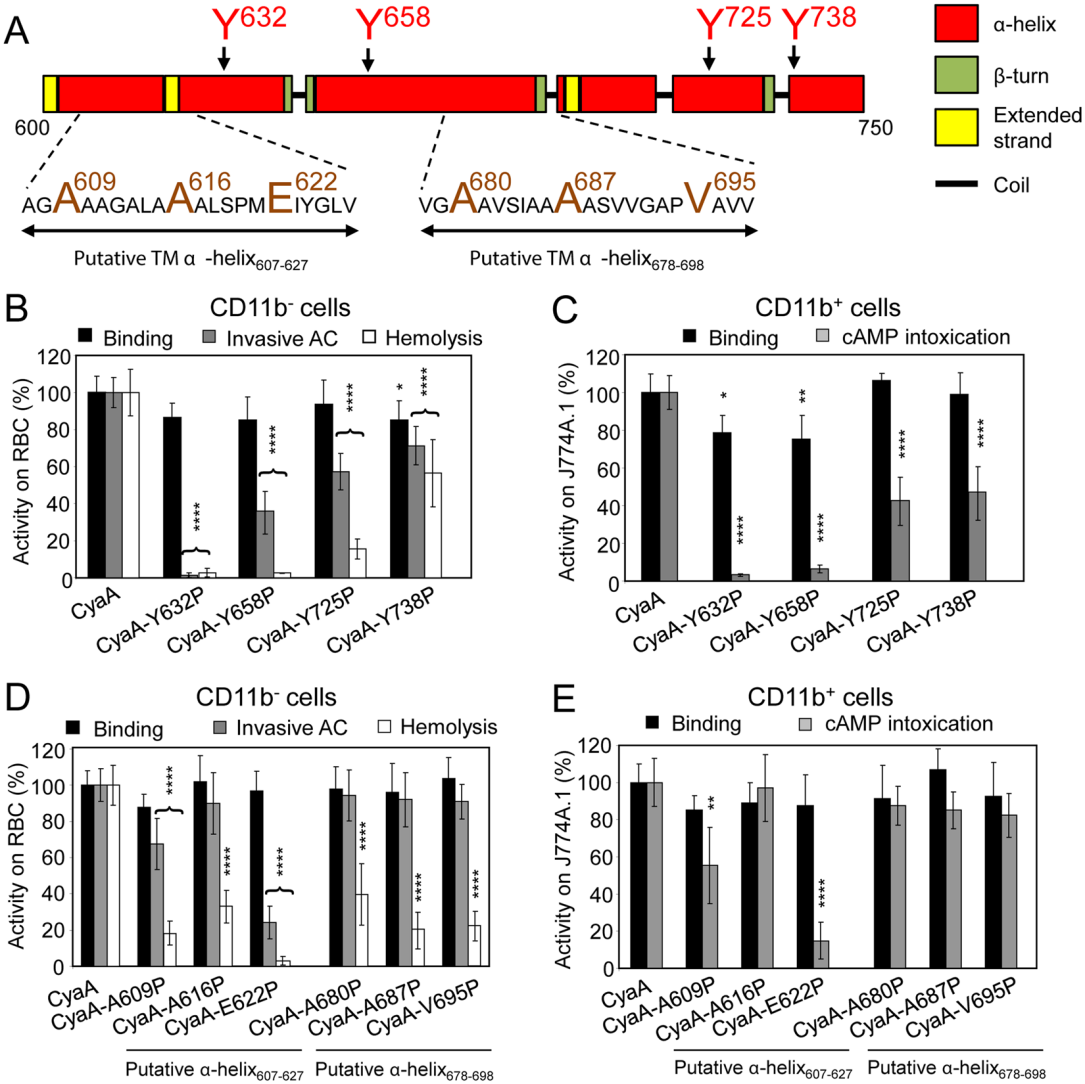
α-helical structures located between residues 600 to 750 of CyaA participate in AC domain translocation and pore formation. (**A**) Schematic secondary structure of the segment 600 to 750 of CyaA with two putative transmembrane (TM) α-helices predicted by Eisenberg method^64^ and located between residues 607 to 627 and 678 to 698. The amino acid residues selected for substitution are enlarged and colored. (**B** to **E**) Biological activities of intact CyaA or its mutant variants were analyzed using CD11b^−^ sheep erythrocytes (**B**,**D**) and CD11b^+^ J774A.1 mouse macrophages (**C**,**E**). Preparations and analyses of samples were performed as in the legend to Fig. 1. All activities are expressed as percentages of intact CyaA activity and represent average values ± standard deviations from at least three independent determinations performed in duplicate with two different toxin preparations. Significant differences are indicated by asterisks (*p < 0.05; **p < 0.01; ***p < 0.001; ****p < 0.0001).

Secondary structure models indicated that the Y632, Y658, Y725 and Y738 residues are predicted to be part of long α-helical segments within the CyaA segment consisting of residues 600 to 750 (Fig. 3A). We therefore examined the impact of substitution of these tyrosine residues by helix-breaking proline residues. As shown in Fig. 3B, the Y632P substitution abolished both the AC domain translocation capacity, as well as the hemolytic activity of the toxin on sheep erythrocytes. The Y658P, Y725P and Y738P substitutions then reduced the specific AC domain translocating capacity only in part (e.g. by ~62, ~43 and ~29%), but the Y658P ablated the hemolytic activity. In contrast, the toxin Y725P and Y738P substitutions had a milder effect, reducing the specific hemolytic activity by ~86% and ~44%, respectively. At the same time, however, all four proline substitutions affected binding of the CyaA mutants to erythrocytes only slightly, if at all (Fig. 3B). Moreover, a similar pattern of cell binding and AC translocating capacities of the four Tyr > Pro CyaA mutants was also observed with CD11b^+^ J774A.1 cells, to which CyaA binds through the CR3 receptor (Fig. 3C, Supplementary Fig. S1)^24, 25^. Indeed, helix-preserving alanine substitutions of the same tyrosine residues had no impact on the activities of CyaA within target membranes (Supplementary Fig. S2). These results suggest that rather than being part of CRAC motifs, the aromatic side chains of the tyrosine residues 632, 658, 725 and 738 play a structural role in α-helical segments that are involved in membrane translocation of the AC domain and in formation of CyaA pores.

**Figure 4:**
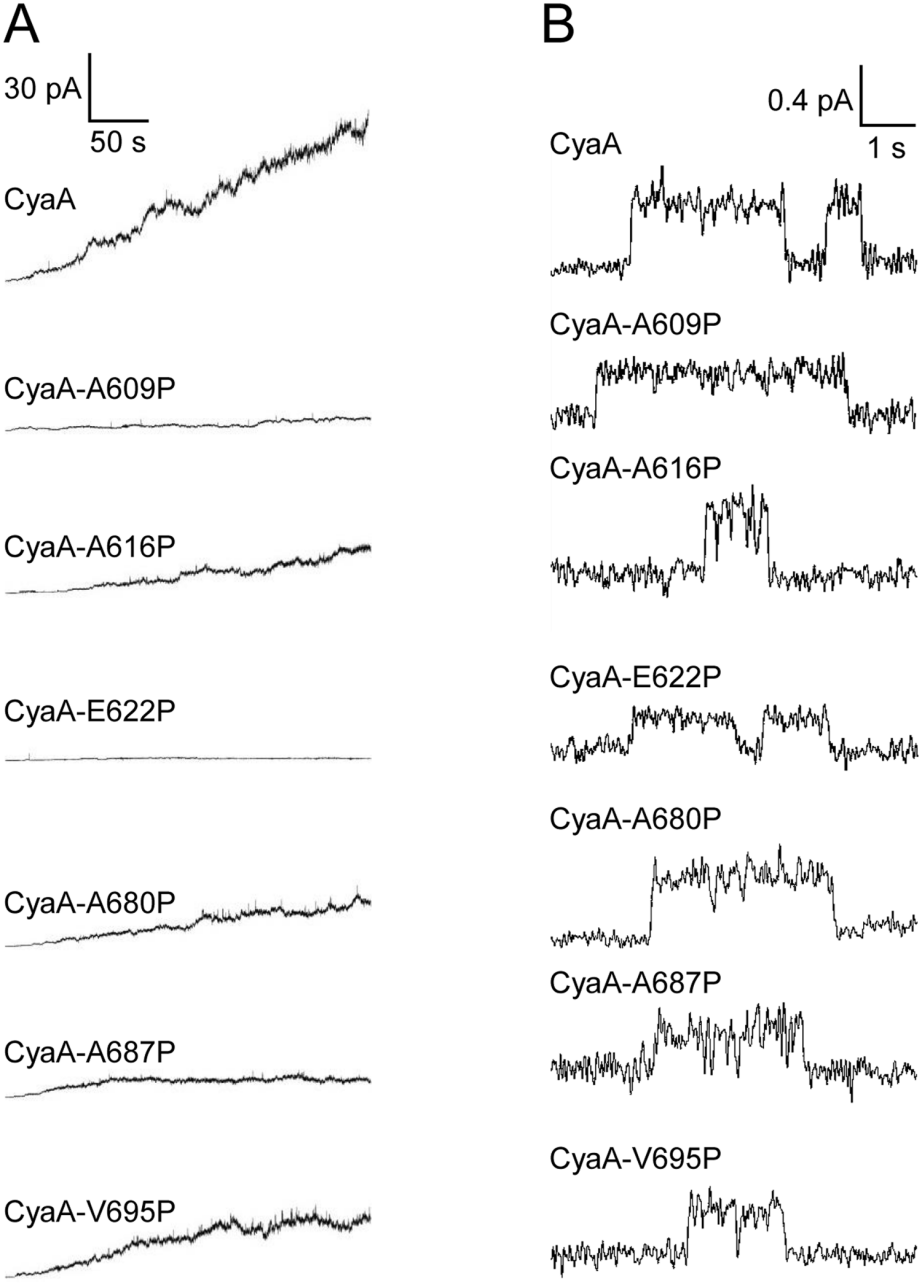
Membrane activity and conductance of single pores is affected by proline substitutions in putative transmembrane α-helices. (**A**) Overall membrane activities of intact CyaA and its mutant variants on asolectin/decane:butanol (9:1) membranes. The applied membrane potential was −50 mV and the temperature was 25 °C. (**B**) Detail of most frequent conductance states on asolectin/decane:butanol (9:1) membranes (filtered at 10 Hz). The presented events were acquired on several different asolectin membranes for each toxin. Measurement conditions: 150 mM KCl, 10 mM Tris-HCl (pH 7.4), 2 mM CaCl_2_, toxin concentration 1 nM, transmembrane potential −50 mV.

The tyrosine residues Y632 and Y658 are located between two putative transmembrane α-helices, predicted to form between residues 607 to 627 (α-helix_607-627_) and 678 to 698 (α-helix_678-698_), while the Y725 and Y738 residues are C-terminally adjacent to the α-helix_678-698_ (Fig. 3A). Previously, devastating effects of deletions destroying these segments (Δ615-655 and Δ663-688) indicated that this region of CyaA molecule may play an important role in membrane activities of the toxin^49^. Moreover, helix-breaking A616P or A687P substitutions within these segments reduced the hemolytic activity of the Hly moiety of CyaA^50^. To verify that these segments form long α-helical structures, we introduced helix-breaking proline residue substitutions at the beginning, in the center and at the end of each of the two predicted α-helices (Fig. 3A). As summarized in Fig. 3D,E, the introduction of proline residues had no or only a negligible effect on the capacity of the mutant toxins to bind cells. Moreover, the binding to CD11b^+^ J774A.1 cells could be blocked by the competing CD11b-specific antibody M1/70 (Supplementary Fig. S1)^24, 25^. However, the substitution of the A609 and E622 residues affected both the capacity of CyaA to translocate the AC domain into cells (Fig. 3D,E) and to form the hemolytic pores within erythrocyte membrane (Fig. 3D). In contrast, substitution of the glutamate 622 by residues that allow formation of an α-helical structure, such as by a neutral glutamine or an oppositely charged lysine residue, had no (CyaA-E622Q), or only a small (CyaA-E622K, ~25–30% decrease) impact on the specific membrane translocation and hemolytic capacities of the toxin, respectively (Supplementary Fig. S3). The proline substitutions of the hydrophobic residues within the α-helices (e.g. A616P, A680P, A687P and V695P) then rather selectively reduced only the specific hemolytic activity of the mutant toxins (Fig. 3D). In line with that, all these proline substitutions affected the pore-forming capacity of the toxin in artificial planar lipid bilayers made of 3% asolectin (Table 1 and Fig. 4). As summarized in Table 1 and Fig. 4, compared to the most frequent single pore conductance of ~8.7 pS, observed for pores formed by intact CyaA, the pores formed by the toxins carrying the proline substitutions exhibited a reduced conductance (e.g. ~6.4 pS for CyaA-A609P, ~5.4 pS for CyaA-E622P, ~6.1 pS for CyaA-A687P and ~6.9 pS for CyaA-V695P). As documented by the number of plus signs in Table 1, and by the representative activity recordings in Fig. 4A, the overall membrane activity (i.e. compounded contributions of pore conductance, pore lifetime, and of the specific frequency of formation of pores) of these proline mutants of CyaA was reduced. In particular, the pores formed by CyaA with proline substitutions within the α-helix_678-698_ were less stable and exhibited shorter mean lifetimes, ranging from 0.74 to 0.88 s, as compared to the mean lifetime of ~1.64 s observed for intact CyaA pores. The predicted α-helix_607-627_ thus appears to be importantly involved in both AC domain delivery and toxin pore formation, while the α-helix_678-698_ appears to be exclusively involved in the pore-forming activity of CyaA.

**Table 1:** Activities of different CyaA mutants on black lipid membranes.

	Most frequent single pore conductance (pS)^a^	Pore lifetime τ (s)^b^	Overall membrane activity^c^
CyaA	8.7 ± 1.8	1.64 ± 0.14	+++
CyaA-A609P	6.4 ± 1.4	1.61 ± 0.42	+
CyaA-A616P	9.6 ± 2.2	1.64 ± 0.23	++
CyaA-E622P	5.4 ± 1.3	1.63 ± 0.51	+/−
CyaA-A680P	8.8 ± 1.7	0.88 ± 0.09	++
CyaA-A687P	6.1 ± 1.7	0.74 ± 0.10	+
CyaA-V695P	6.9 ± 1.8	0.86 ± 0.37	++
CyaA-Y940F	7.3 ± 1.6	2.01 ± 0.45	+++
CyaA-Y940A	8.0 ± 1.6	1.40 ± 0.18	+/−
CyaA-Y940P	6.3 ± 1.3	1.75 ± 0.76	+/−

^a^Single-pore conductance of intact CyaA and its mutant variants (1 nM) was determined in 150 mM KCl, 10 mM Tris-HCl and 2 mM CaCl_2_ (pH 7.4) at 25 °C and membrane potential −50 mV. The average values ± S.D. (half width at half maximum) are shown.

^b^For lifetime determination, the kernel density estimation of dwell times (of ~700 individual pore openings) was fitted with a double-exponential function. The error estimates of lifetimes were obtained by bootstrap analysis. We show only lifetime > 500 ms.

^c^The overall membrane activity was detected after 5 minutes incubation of the membranes with individual proteins at 1 nM concentration. The number of plus signs refers to the overall conductance of the membrane/cm^2^ induced by the various CyaA proteins under these conditions in asolectin membranes and reflects the conductance (size), the lifetime, and the specific frequency of formation of pores by the various CyaA constructs.

### The aromatic side chain of tyrosine residue 940 plays a key role in penetration of CyaA into target membrane

**Figure 5:**
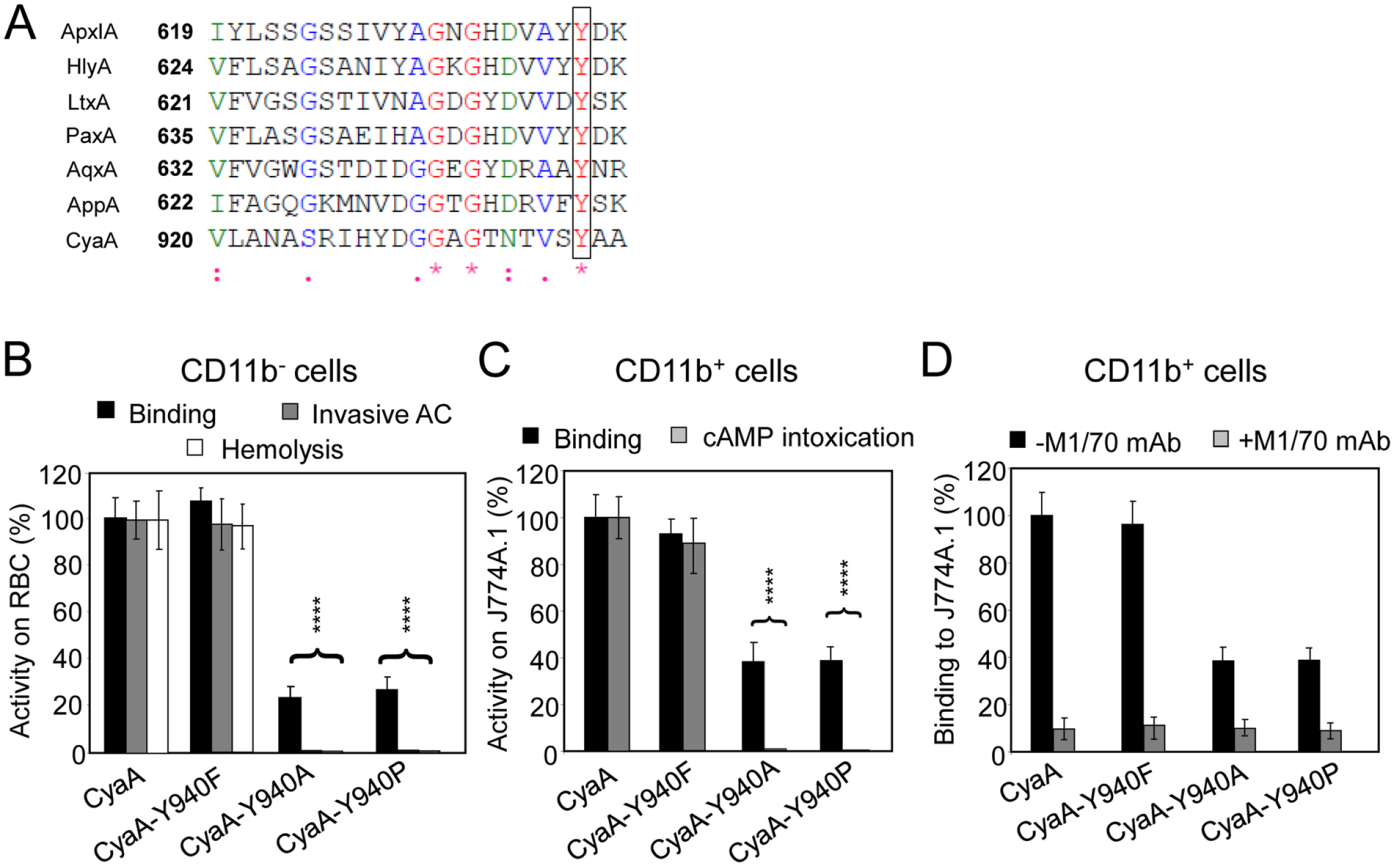
Y940A and Y940P substitutions substantially reduce binding of CyaA to cells. (**A**) ClustalW sequence alignment of a partial sequence of the acylated domain of CyaA and corresponding sequences of related RTX toxins. ApxIA, *Actinobacillus pleuropneumoniae* (uniprot P55128); HlyA, *Escherichia coli* (uniprot Q8G9Z4); LtxA, *Aggregatibacter actinomycetemcomitans* (uniprot P16462); PaxA, *Pasteurella aerogenes* (uniprot Q9RCG8); AqxA, *Actinobacillus equuli* (uniprot Q8KWZ9); AppA, *Kingella kingae* (uniprot F5S905); and CyaA, *Bordetella pertussis* (uniprot code P0DKX7). The highly conserved tyrosine residues (Y940 in CyaA) are highlighted by a black frame, * identity,: strongly similar,. weakly similar. (**B**,**C**) Biological activities of intact CyaA or its mutant variants were analyzed using CD11b^−^ sheep erythrocytes (**B**) and CD11b^+^ J774A.1 mouse macrophages (**C**). Preparations and analyses of samples were performed as in the legend to Fig. 1. Activities are expressed as percentages of intact CyaA activity and represent average values ± standard deviations from at least three independent determinations performed in duplicate with two different toxin preparations. Significant differences are indicated by asterisks (****, p < 0.0001). (**D**) To block the CR3 receptor of CyaA, J774A.1 cells were preincubated for 30 minutes on ice with 5 µg/ml of the CD11b-specific monoclonal antibody M1/70 (Pharmingen) prior to addition of the CyaA variants (1 µg/ml). J774A.1 binding activities are expressed as percentages of intact CyaA binding activity and represent average values ± standard deviations from three independent determinations.

The Y940 residue is located in a predicted β-strand structure within the acylated domain of CyaA (Fig. 1A) and it appears to be highly conserved within the family of RTX toxins (Fig. 5A). To examine its role in activities of CyaA, we replaced Y940 with phenylalanine, alanine and proline residues. As verified by tandem mass spectrometry and documented in Table 2, the substitutions of the Y940 residue had no impact on the capacity of the co-expressed CyaC transacylase to recognize the acylated domain of proCyaA and to accomplish full fatty-acylation of the K860 and K983 residues, of which the latter is essential for the capacity of CyaA to bind and penetrate target membranes^13–15, 51^. The K860 and K983 residues of the CyaA-Y940F, CyaA-Y940A and CyaA-Y940P constructs were acylated by palmitoyl (C16:0) and palmitoleyl (cisΔ9 C16:1) residues to a comparable extent and at similar ratios as in intact CyaA. However, by difference to the CyaA-Y940F construct, which exhibited the same specific toxin activity on cells as intact CyaA, the CyaA-Y940A and CyaA-Y940P proteins exhibited a two- to three-fold reduced specific cell-binding capacity (Fig. 5B,C). Moreover, these constructs were essentially unable to translocate the AC domain across the membrane of target cells (Fig. 5B,C) and were largely impaired in the capacity to form the hemolytic pores within erythrocyte membrane (Fig. 5B). This was most likely not due to a defect of folding of the RTX domain that is required for CR3 receptor binding, as the reduced binding of the Y940A and Y940P constructs to CD11b^+^ J774A.1 could still be blocked by the CD11b-specific competing antibody M1/70 (Fig. 5D)^24, 25^.

**Table 2:** Acylation status of different CyaA mutants.

Protein^a^	K860		K983	
	Palmitoleyl (%)^c^	Palmitoyl (%)^c^	Palmitoleyl (%)^c^	Palmitoyl (%)^c^
proCyaA^b^	0.2	0	3.6	0.8
CyaA	44.3	45.9	55.9	22.1
CyaA-Y940F	38.1	57.8	41.6	20.8
CyaA-Y940A	39.3	55.8	56.8	22.4
CyaA-Y940P	42.9	50.9	55.9	19.8

^a^All CyaA proteins were produced in the *E. coli* strain XL1-Blue and purified close to homogeneity.

^b^The proCyaA variant was expressed in the absence of the CyaC acyltransferase.

^c^Percentage distribution of fatty acid modification of the ε-amino groups of the K860 and K983 residues by palmitoleyl (cisΔ9 C16:1) and palmitoyl (C16:0) chains. Average values are derived from determinations performed with two different toxin preparations. The remaining Lys860 and Lys983 residues to 100% are non-acylated.

**Figure 6:**
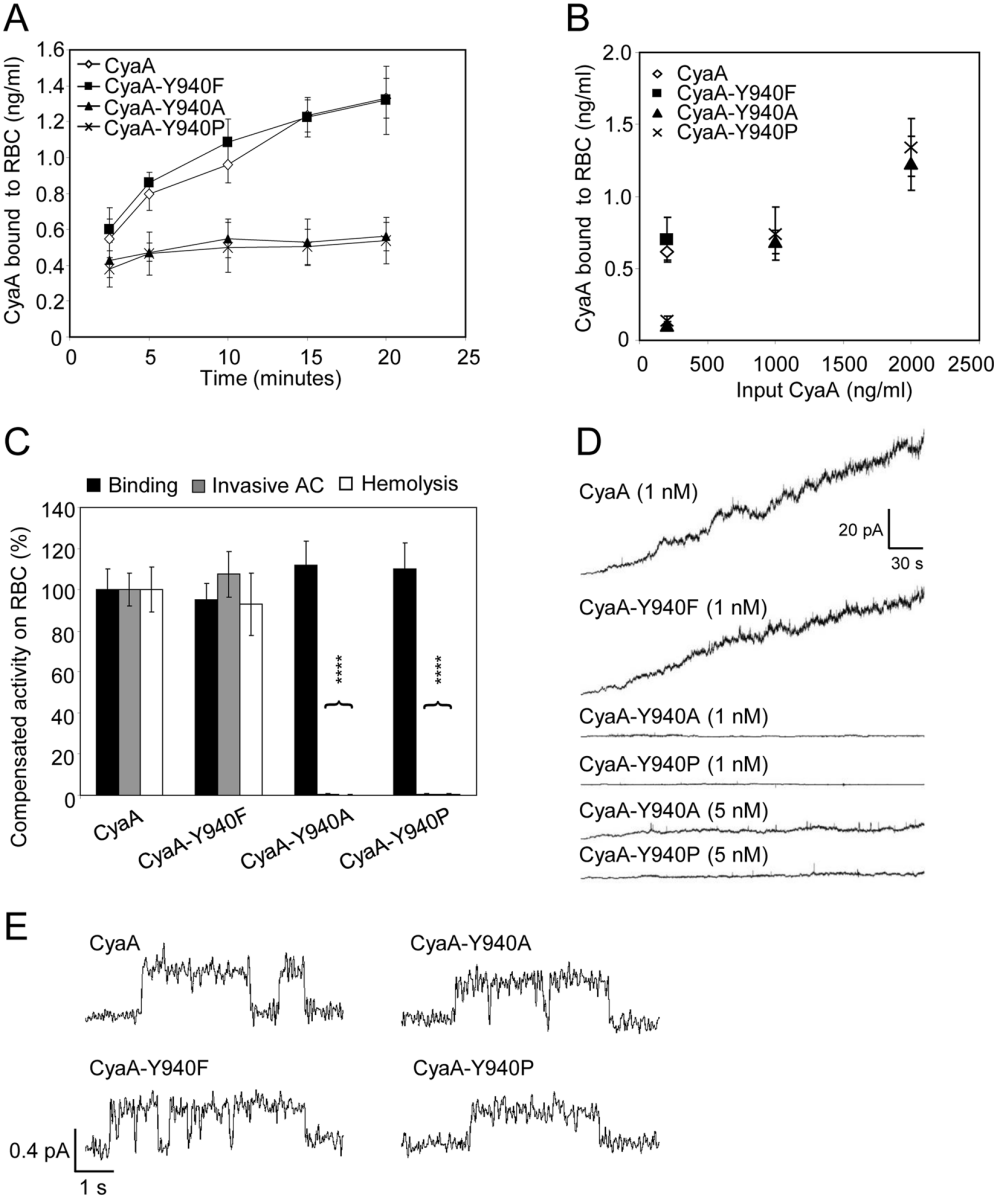
The Y940A and Y940P substitutions impair membrane insertion and AC domain translocating and pore forming capacities of CyaA. (**A**) Sheep erythrocytes (5 × 10^8^/ml) were incubated with 1 µg/ml (5 nM) of CyaA variants and binding was determinate after 2.5, 5, 10, 15 and 20 minutes as membrane associated AC activity as described in the Methods section. Binding activities represent average values ± standard deviations from three independent determinations. (**B**) Sheep erythrocytes were incubated as above with the indicated concentrations of the enzymatically active CyaA-derived proteins (input CyaA) for 30 minutes, washed, and the amount of cell-associated AC enzyme activity was determined. (**C**) To compensate for the reduced cell binding activity, the concentration of the CyaA-Y940A and CyaA-Y940P proteins was increased to 1 µg/ml (5 nM), as compared with 200 ng/ml (1 nM) for intact CyaA or CyaA-Y940F, allowing to achieve binding of equal amounts of each protein per ml of erythrocytes (~0.6 to 0.7 ng/ml, see panel B). Binding and invasive AC of intact CyaA was taken as 100%. For comparison of hemolytic activity, the concentration of CyaA-Y940A and CyaA-Y940P was increased to 25 µg/ml, compared to 5 µg/ml for intact CyaA or CyaA-Y940F. Hemolytic activity was measured after 6 hours by photometric determination (A_541nm_) and activity of intact CyaA was taken as 100%. Activities represent average values ± standard deviations from three independent determinations performed in duplicate. (**D**) Overall membrane activities of intact CyaA and its mutant variants on asolectin/decane:butanol (9:1) membranes. The applied membrane potential was −50 mV and the temperature was 25 °C. (**E**) Detail of most frequent conductance states on asolectin/decane:butanol (9:1) membranes (filtered at 10 Hz). The presented events were acquired on several different asolectin membranes for each toxin. Measurement conditions: 150 mM KCl, 10 mM Tris-HCl (pH 7.4), 2 mM CaCl_2_, toxin concentration 1 nM, transmembrane potential −50 mV.

Kinetic analysis of binding of the CyaA-Y940F/A/P toxin variants to erythrocytes revealed that while the amount of cell-bound CyaA-Y940F protein was increasing in time, as for intact CyaA (Fig. 6A), the initial low amount of cell-adsorbed CyaA-Y940A and CyaA-Y940P proteins hardly increased at all over the time of incubation with cells (Fig. 6A). This indicates that the capacity of the CyaA-Y940A and CyaA-Y940P constructs to irreversibly insert into the erythrocyte membrane, and thereby shift the cell association-dissociation equilibrium over time, was importantly impaired. Therefore, the specific AC translocating and pore-forming capacities of CyaA-Y940A and CyaA-Y940P constructs were compared to those of intact CyaA and CyaA-Y940F under conditions where equal CyaA protein amounts were bound per erythrocyte. This was achieved by increasing the input concentration of CyaA-Y940A/P variants in the binding assay approximately five-fold over the amount of intact CyaA or CyaA-Y940F (Fig. 6B). As shown in Fig. 6C, even under such conditions the CyaA-Y940A and CyaA-Y940P proteins still exhibited an almost nil AC domain translocation and pore-forming capacity on erythrocytes. In line with that, and despite the presence of an intact pore-forming domain, the CyaA-Y940A and CyaA-940P proteins exhibited a particularly low overall membrane activity also on artificial lipid bilayers (Table 1 and Fig. 6D). As further shown in Table 1 and Fig. 6E, the typical single pore conductance (8.0 ± 1.6 pS) and the mean lifetime (1.40 ± 0.18 s) of pores formed by the CyaA-Y940A variant were comparable to those of intact CyaA (8.7 ± 1.8 pS and 1.64 ± 0.14 s, respectively), but the CyaA-Y940A mutant formed pores with a much lower frequency than intact CyaA (Fig. 6D). The particularly rarely opening CyaA-Y940P pores exhibited a partially reduced conductance (6.3 ± 1.3 pS), with mean lifetime again comparable to those of intact CyaA (1.75 ± 0.76 s, Table 1 and Fig. 6E). These data, hence, reveal that the aromatic ring of the side chain of the highly conserved Y940 residue plays a key structural role in the CyaA molecule and its presence is essential for the initial interaction of CyaA with the lipid bilayer of the membrane, triggering proper insertion of crucial toxin segments and translocation of the AC domain and toxin pore formation.

## Discussion

We show here that the conserved tyrosine 940 residue of the putative CRAC motif in the acylated domain of CyaA plays a central structural role in membrane insertion and penetration of the toxin. Analysis of the functional role of the putative CRAC motifs in the Hly moiety of CyaA then revealed that these are not involved in cholesterol binding and that the C-terminal segment of the hydrophobic domain of CyaA consists of long α-helical structures that play a key role in toxin activities within target membranes.

We have initiated this work as our previous observations indicated that presence and relative content of cholesterol within membrane of CD11b^+^ macrophages determines the capacity of the membrane-inserted CyaA toxin to complete the translocation of the AC domain across the bilayer of membrane lipid microdomains (rafts) into target cell cytosol^39^. The herein presented results, however, reveal that CyaA does not directly bind cholesterol through the putative CRAC motifs within its Hly moiety. In line with previous observation that CyaA can insert into cholesterol depleted membranes^39^, these results suggest that interaction of the toxin with cholesterol is not involved in the initial interaction with the lipid bilayer prior to toxin incorporation into the plasma membrane of target cells. This distinguishes the mechanism of membrane penetration of CyaA from that of other related RTX cytolysins, such as the *A. actinomycetemcomitans* LtxA and *E. coli* HlyA, which possess *bona fide* cholesterol-binding sites and bind cholesterol with high affinity^41–43, 52^. Three motifs have, indeed, been previously implicated in cholesterol binding to transmembrane proteins, namely CRAC, CARC (inverted CRAC with the consensus sequence R/K-(X)(1–5)-Y/F-(X)(1–5)-L/V) and a cholesterol consensus motif^53^. The stringency of the consensus of the most well-known CRAC motif appears to be low and not all putative CRAC motifs bind cholesterol^54^. This appears to be also the case of the here-analyzed four predicted putative CRAC sites identified within the membrane-interacting Hly moiety of CyaA. Their key tyrosine residues (Y632, Y658, Y725 and Y738) could be substituted with phenylalanine or alanine residues without causing any decrease of membrane penetration and translocation activity of the toxin (*c.f*. Fig. 1 and Supplementary Fig. S2). In contrast, substitutions of these tyrosine residues by helix-breaking proline residues importantly reduced the activities of the toxin in target membranes (*c.f*. Fig. 3B,C). Hence, rather than being part of the CRAC motifs, the tyrosine residues 632, 658, 725 and 738 play a structural role in formation of the long α-helices that can be predicted in the segment comprising residues 600 to 750 of CyaA. That CyaA does not contain functional CRAC motifs that would bind cholesterol is also indicated by the observation that as high as 5 µM free cholesterol did not inhibit the capacity of CyaA to penetrate target cells. In contrast, the hemolytic activity of the related ApxIA toxin was affected already at 0.5 nM concentration of free cholesterol, while the structurally related ergosterol had no effect. It is thus plausible to speculate that by difference to CyaA, the ApxIA toxin recognizes and binds cholesterol through its consensus CRAC-CARC “mirror“ motif^55^ that is not present in CyaA (Supplementary Fig. S4). The cholesterol content of the membrane would thus modulate membrane insertion of CyaA^40^ and translocation of its AC domain^39^ in an indirect way, determining the propensity of lipids to lateral phase separation. Cholesterol would then increase the propensity of formation of liquid-ordered (lo) phase at the expense of liquid-disordered (ld) phase, thus lowering the energy barrier for AC domain translocation across lipid bilayer^56, 57^.

**Figure 7:**
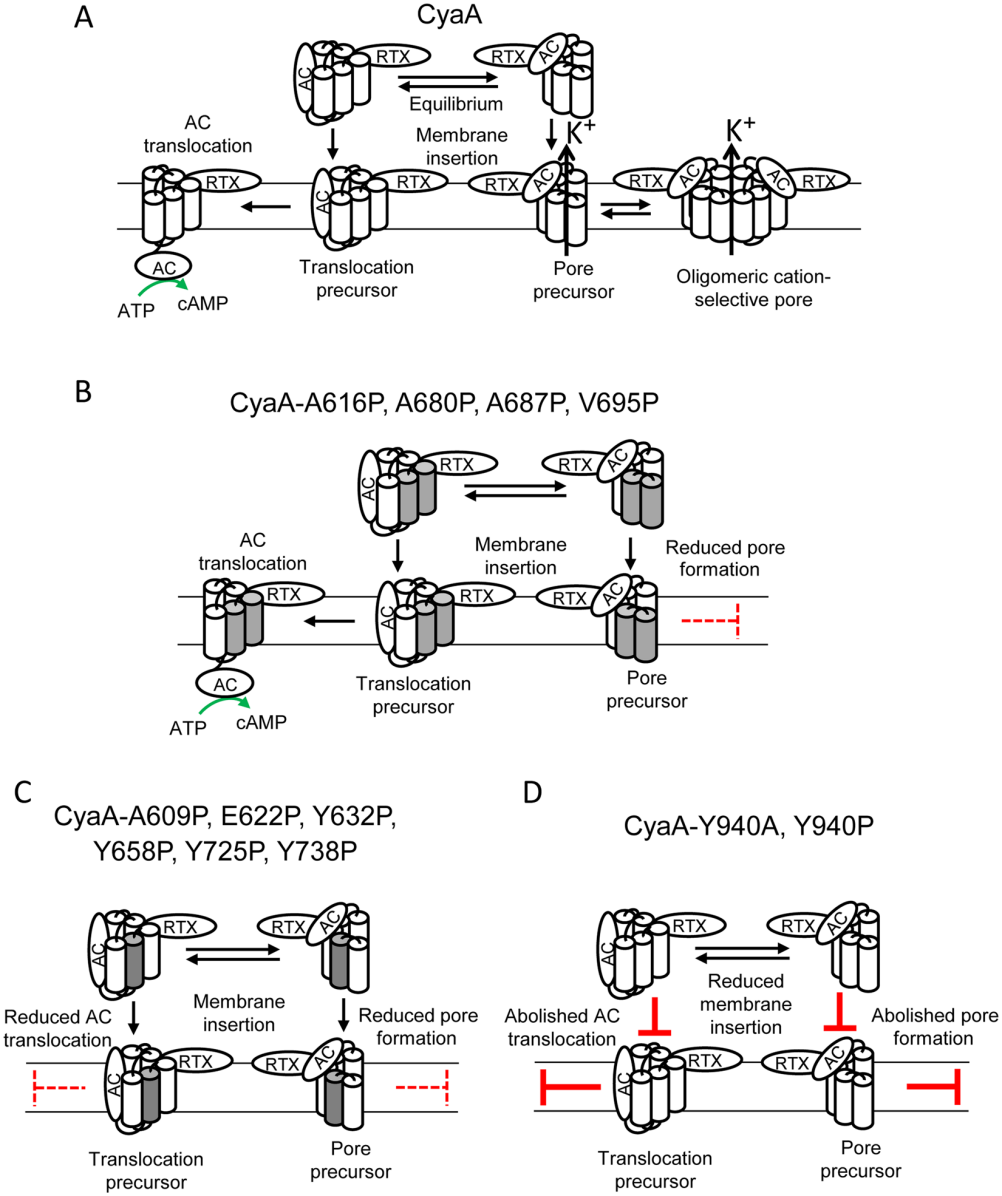
Schematic model of CyaA interaction with target membrane. (**A**) In solution, two conformational isomers of CyaA co-exist in equilibrium that, upon membrane insertion, yield either a translocation precursor competent for subsequent translocation of the AC domain across target membrane, or a pore precursor competent for K^+^ efflux and involved in formation of oligomeric CyaA pores^2, 10, 32–35^. (**B**) Proposed model for the membrane interaction of the CyaA-A616P, CyaA-A680P, CyaA-A687P and CyaA-V695P mutants. Helix-breaking A616P substitution (A680P, A687P and V695P) within the predicted transmembrane α-helix_607-627_ as well as three proline substitutions within the predicted transmembrane α-helix_678-698_ selectively reduced the pore-forming activity of CyaA, but did not affect the capacity of CyaA to translocate the AC domain across the membrane. The putative transmembrane α-helices 607 to 627 and 678 to 698 are marked in gray. (**C**) Proposed model for the membrane interaction of the CyaA-A609P, CyaA-E622P, CyaA-Y632P, CyaA-Y658P, CyaA-Y725P and CyaA-Y738P mutants. The helix-breaking A609P and E622P substitutions within the predicted transmembrane α-helix_607-627_ reduced both AC domain translocation and CyaA pore formation similarly as the proline substitutions of the tyrosine residues Y^632^, Y^658^, Y^725^ and Y^738^, located within long α-helical structures and in the proximity of the putative transmembrane α-helices 607 to 627 and 678 to 698. The putative transmembrane α-helix_607-627_ is marked in gray. (**D**) Proposed model for the membrane interaction of the CyaA-Y940A and CyaA-Y940P mutants. The Y940A and Y940P substitutions substantially reduced specific capacity of CyaA to bind target plasma membrane and abolished AC domain translocation and formation of pores.

The here-analyzed segment of the pore-forming region, where four out of the five predicted putative CRAC motifs of the Hly are located, comprises also two predicted transmembrane α-helices (residue 607 to 627 and 678 to 698). Data presented here suggest that helix 607 to 627 is involved in translocation of the AC domain and in the parallel formation of CyaA pores. Indeed, helix-breaking proline substitutions within the putative transmembrane α-helix_607-627_ substantially reduced the pore-forming capacity of CyaA. The A609P and E622P substitutions also decreased the AC domain translocation capacity of the toxin (Fig. 7). On the other hand, all three proline substitutions within the predicted transmembrane α-helix_678-698_ selectively affected only the pore-forming activity of CyaA and altered the most frequent lifetimes or pore conductances of the formed CyaA pores. However, these substitutions did not affect the capacity of CyaA to translocate the AC domain across the membrane (Fig. 7). Similar dissociation of the pore-forming and AC translocating activities of CyaA has, indeed, been previously observed upon introduction of certain residue substitutions within two of three previously identified transmembrane α-helices^32, 35, 58^, predicted to form between residues 502 to 522, 529 to 549 and 565 to 591 of the pore-forming domain. The E516Q substitution selectively increased and the E570Q substitution selectively decreased the propensity of CyaA to form pores. Both substitutions, however, had no impact on the AC domain translocating capacity of the toxin^32^. Similar membrane activity dissociating effects were observed for diverse charge-altering or helix-breaking substitutions within the five thus far identified and mutagenized segments of the pore-forming domain. This would indicate that all these putative transmembrane α-helices cooperate in translocation of the AC domain and in formation of the CyaA pores within target membranes.

The molecular details of the mechanism of penetration of RTX toxins into target cell membranes and the role of their acylated domains in this process remain, however, poorly understood. Contrary to what one would expect, the fatty acyl modification of CyaA on lysine residues K860 and K983 does not appear to be *per se* essential for penetration of the toxin into a naked lipid bilayer membrane^31^. Rather, the fatty acyl chains linked to ε-amino groups of K860 and K983 residues appear to play a structural role in folding of the N-terminal (~600 residue-long) non-RTX moiety of CyaA^59^. By affecting the aggregation status of CyaA and the folding of its pore-forming domain, the acylation of K860 and K983 residues appears to enable an irreversible insertion of the hydrophobic domain of CyaA into cellular membrane, thus stabilizing the interaction of the toxin with its CR3 receptor on target cell membrane^15^. Moreover, the here-observed effects of the alanine and proline substitutions of the Y940 residue of the fifth putative CRAC motif reveal that the acylated domain plays a structural role in toxin activities within target membranes. The CyaA-Y940A and CyaA-Y940P proteins were largely impaired in their capacity to tightly associate with target cell membrane and were unable to translocate the AC domain or form pores across cellular membrane. In contrast, the Y940F construct with the phenolic group of the tyrosine side chain replaced by a simple benzene ring was fully active. This strongly suggests that the aromatic ring of the side chain of tyrosine 940 within the acylated domain plays a central role in formation of a structure that plays a crucial role in initiation of insertion and in proper positioning of the transmembrane segments of CyaA within target cell membrane. This would, indeed, go well with the recent identification of a conserved lysine/arginine residue cluster within the acylated domain that was predicted to participate in interaction of the acylated domain of CyaA with target cell membrane^60^.

## Methods

### Construction, production and purification of CyaA and ApxIA proteins

The pCACT3 plasmid was used for co-expression of *cyaC* and *cyaA* genes allowing production of recombinant CyaC-activated CyaA in *E. coli*.^49^. Oligonucleotide-directed PCR mutagenesis was used to construct pCACT3-derived plasmids for expression of CyaA mutant variants. Intact CyaA and its mutant variants were produced in the *E. coli* strain XL1-Blue (Stratagene) transformed with appropriate pCACT3-derived constructs. The cells were grown at 37 °C in MDO medium (yeast extract, 20 g/l; glycerol, 20 g/l; KH_2_PO_4_, 1 g/l; K_2_HPO_4_, 3 g/l; NH_4_Cl, 2 g/l; Na_2_SO_4_, 0.5 g/l; thiamine hydrochloride, 0.01 g/l) supplemented with 150 μg/ml of ampicillin, induced at OD_600_ = 0.6 with 1 mM isopropyl 1-thio-β-D-galactopyranoside (IPTG), and grown for an additional 4 hours. Then the cells were collected, disrupted by ultrasound, and the insoluble cell debris was extracted with 8 M urea in 50 mM Tris-HCl (pH 8.0) (TU buffer) containing 0.2 mM CaCl_2_. The CyaA proteins were purified by ion-exchange chromatography on DEAE-Sepharose followed by hydrophobic chromatography on phenyl-Sepharose as previously described^3^. For expression of the acylated ApxIA toxin^48^, the *E. coli* strain BL21 (λDE3) was transformed with the pET28b-apxIC-apxIA plasmid, harboring the *apxIC* and *apxIA* genes^61^. The cells were grown at 37 °C in MDO medium supplemented with 60 μg/ml of kanamycin, induced at OD_600_ = 0.6 with IPTG, and grown for an additional 4 hours. The cells were harvested by centrifugation, disrupted by ultrasound, and the insoluble cell debris was extracted with TU buffer. The urea extract was loaded on an Ni-NTA Sepharose column (GE Healthcare) that was subsequently washed with TU buffer containing 40 mM imidazole. The ApxIA protein was eluted from the column by TU buffer supplemented with 200 mM imidazole that was removed by Sephadex G-25 (GE Healthcare) column chromatography. Concentrations of the purified CyaA and ApxIA proteins were determined by the Bradford assay (Bio-Rad). The integrity of all proteins was systematically controlled by SDS/PAGE (not shown).

### Cell binding, cell invasive and hemolytic activities on sheep erythrocytes

AC enzymatic activities were measured in the presence of 1 µM calmodulin as previously described^62^. One unit of AC activity corresponds to 1 µmol of cAMP formed per minute at 30 °C, pH 8.0. Hemolytic activity was measured by determining the hemoglobin release in time upon toxin incubation with washed sheep erythrocytes (5 × 10^8^/ml) as previously described^6^. Cell invasive AC was measured as previously described^3^, by determining the AC protected against externally added trypsin upon internalization into sheep erythrocytes. Erythrocyte binding of the toxins was determined as previously described^3^. Activity of intact CyaA was taken as 100%.

### Binding and cAMP elevation of CyaA on J774A.1 cells

Murine monocytes/macrophages J774A.1 (ATCC, number TIB-67) were cultured at 37 °C in a humidified air/CO_2_ (19:1) atmosphere in RPMI medium supplemented with 10% (v/v) heat-inactivated fetal bovine serum, penicillin (100 i.u./ml), streptomycin (100 µg/ml) and amphotericin B (250 ng/ml). Prior to assays, RPMI was replaced with D-MEM medium (contains 1.9 mM Ca^2+^) without FCS and the cells were allowed to rest in D-MEM for 1 hour at 37 °C in a humidified 5% CO_2_ atmosphere^16^. J774A.1 cells (10^6^) were incubated in D-MEM with 1 μg/ml of CyaA variants for 30 minutes at 4 °C, prior to removal of unbound toxin by three washes in D-MEM. After the transfer to the fresh tube, the cells were lyzed with 0.1% Triton X-100 for determination of cell-bound AC enzyme activity. For intracellular cAMP assays, 2 × 10^5^ cells were incubated at 37 °C with CyaA for 30 minutes in D-MEM, the reaction was stopped by addition of 0.2% Tween-20 in 100 mM HCl, samples were boiled for 15 minutes at 100 °C, neutralized by addition of 150 mM unbuffered imidazole and cAMP was measured by a competitive immunoassay^3^. Activity of intact CyaA was taken as 100%.

### Liquid chromatography-mass spectrometry (LC-MS) analysis

The CyaA proteins were dissolved in 100 mM 4-ethyl morpholine (pH 8.3) to reach 4 M concentration of urea and digested with trypsin (Promega, modified sequencing grade) at a trypsin:protein ratio of 1:50 for 6 hours at 30 °C. Then the 2nd portion of trypsin was added to a final ratio of trypsin:protein of 1:25 and the reaction was carried out for another 6 hours at 30 °C. When the reaction was complete, the concentration of the resulting peptides was adjusted by 0.1% trifluoroacetic acid (TFA) to 0.1 mg/ml and 3 µl of the sample were injected into the LC-MS system. The LC separation was performed using a desalting column (ZORBAX C18 SB-300, 0.1 × 2 mm) at a flow rate of 40 µl/min (Shimadzu) of 0.1% FA and a separation column (ZORBAX C18 SB-300, 0.2 × 150 mm) at a flow rate of 10 µl/min (Agilent 1200) of water/acetonitrile (MeCN) (Merck) gradient: 0-1 minute 0.2% formic acid (FA), 5% MeCN; 5 minutes 0.2% FA, 10% MeCN; 35 minutes 0.2% FA, 50% MeCN; 40 minutes 0.2% FA, 95% MeCN; 40–45 minutes 0.2% FA, 95% MeCN. A capillary column was directly connected to a mass analyzer. The MS analysis was performed on a commercial solariX XR FTMS instrument equipped with a 15 T superconducting magnet and a Dual II ESI/MALDI ion source (Bruker Daltonics). Mass spectra of the CyaA samples were obtained in the positive ion mode within an m/z range of 250-3000. The accumulation time was set at 0.2 s, LC acquisition was 45 minutes with 5 minutes delay and one spectrum consisted of accumulation of four experiments. The instrument was externally calibrated using Agilent tuning mix, which results in typical mass accuracy below 2 ppm. After the analysis the spectra were processed using DA 4.4 software package (Bruker Daltonics).

### Lipid bilayer experiments

Measurements on planar lipid bilayers (black lipid membranes) were performed in Teflon cells separated by a diaphragm with a circular hole (diameter 0.5 mm) bearing the membrane. The CyaA toxin was diluted in 8 M urea, 50 mM Tris-HCl (pH 8.0) and added into the grounded cis compartment with a positive potential. The membrane was formed by the painting method using soybean lecithin in n-decane–butanol (9:1, vol/vol). Both compartments contained 150 mM KCl, 10 mM Tris-HCl (pH 7.4), and 2 mM CaCl_2_, the temperature was 25 °C. The membrane current was registered by Ag/AgCl electrodes (Theta) with salt bridges (applied voltage, 50 mV), amplified by an LCA-200-100G amplifier (Femto), and digitized by use of a KPCI-3108 card (Keithly). For lifetime determination, approximately 700 of individual pore openings were recorded and the dwell times were determined using QuB software with 10 Hz low-pass filter. The kernel density estimation fitted with a double-exponential function using Gnuplot software. The relevant model was selected by the χ2 value. We show only lifetime > 500 ms (shorter lifetime is not essential for overall membrane activity). The error estimates of lifetimes were obtained by the bootstrap analysis.

### Statistical analysis

Statistical analysis was performed by one-way ANOVA followed by Dunnett’s post-test, comparing all the samples with the control. GraphPad Prism 7.0 (GraphPad Software) was used to perform statistical analysis. Significant differences are indicated by asterisks (*p < 0.05; **p < 0.01; ***p < 0.001; ****p < 0.0001).

## Electronic supplementary material

Supplementary Information
