# Supplementary material for: The conserved tyrosine residue 940 plays a key structural role in membrane interaction of Bordetella adenylate cyclase toxin
Source: Sci Rep. 2017 Aug 24;7:9330. doi: 10.1038/s41598-017-09575-6 (PMC5571180; doi:10.1038/s41598-017-09575-6)
Supplement: Supplementary file 1 — Supplementary Information [file 41598_2017_9575_MOESM1_ESM.pdf]

Supplementary Information

The conserved tyrosine residue 940 plays a key structural role in membrane interaction of *Bordetella* adenylate cyclase toxin

Jiri Masin, Jana Roderova, Adriana Osickova, Petr Novak, Ladislav Bumba, Radovan Fiser, Peter Sebo and Radim Osicka

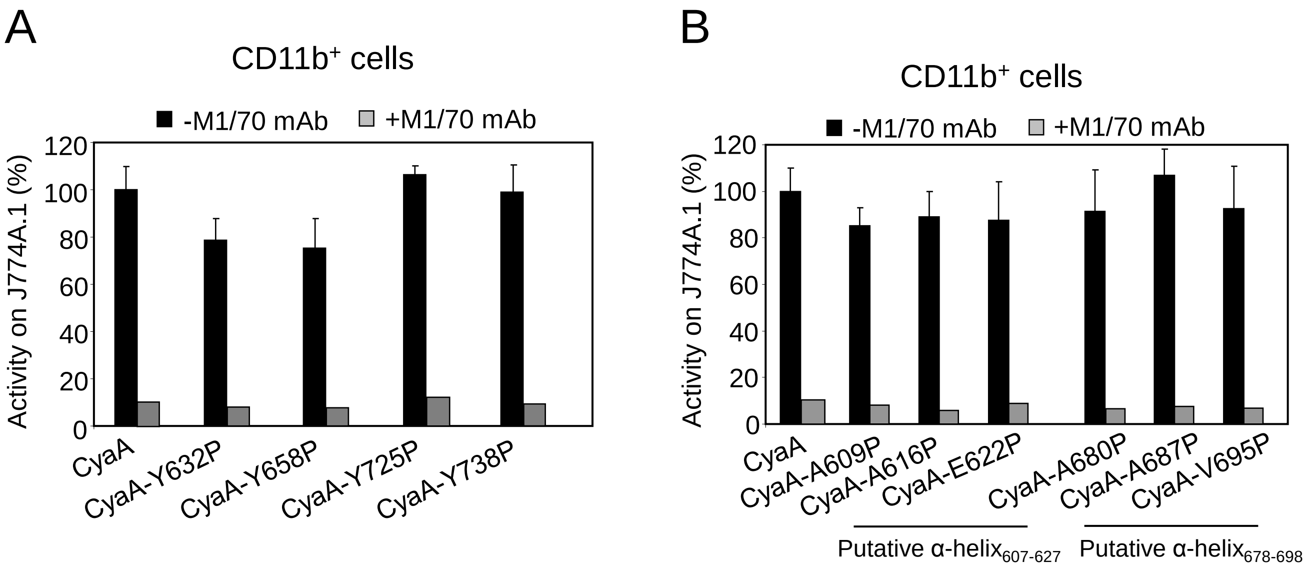

Supplementary Figure S1. Binding of the CyaA mutants to macrophages can be blocked by CD11b-specific antibody. (A, B) Binding of intact CyaA or its mutant variants to J774A.1 cells ($1x10^6$) was determined as the amount of total cell-associated AC enzyme activity upon incubation of cells with 1 μg/ml of the protein for 30 min at 4°C. To block the CR3 receptor of CyaA, J774A.1 cells (10^6/ml) were preincubated for 30 minutes on ice with 5 μg/ml of the CD11b-specific monoclonal antibody M1/70 (Pharmingen) prior to addition of the CyaA variants (1 μg/ml). Activities in the absence of antibody (-M1/70 mAb) are expressed as percentages of intact CyaA activity and represent average values ± standard deviations from at least three independent determinations performed in duplicate with two different toxin preparations. Activities in the presence of antibody (+M1/70 mAb) are expressed as percentages of intact CyaA activity and represent average from two independent determinations performed in monoplicate.

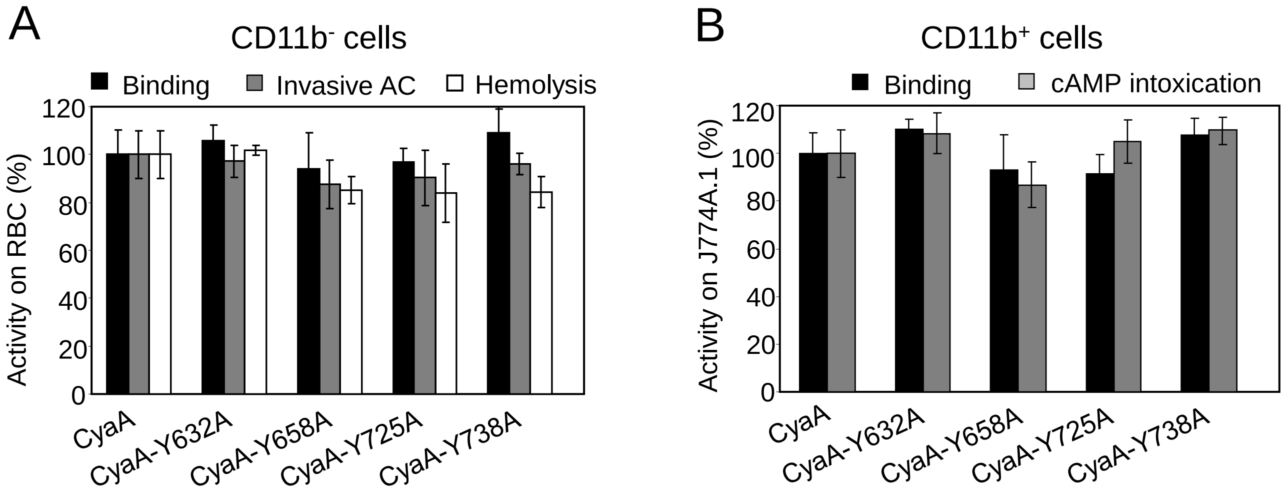

Supplementary Figure S2. Replacement of the key tyrosine residues by alanine residues in the putative CRAC motifs of the hydrophobic domain of CyaA has no significant effect on cell binding, AC domain translocation and pore-forming capacities of toxin variants. (A) Sheep erythrocytes ($5x10^8$/ml) were incubated at 37°C with 1 µg/ml (5 nM) of intact CyaA or its mutant variants and after 30 min, aliquots were taken for determinations of the cell-associated AC activity and of the AC activity internalized into erythrocytes and protected against digestion by externally added trypsin. For determination of hemolytic activity, sheep erythrocytes ($5x10^8$/ml) were incubated at 37°C in the presence of 10 µg/ml (50 nM) of intact CyaA or its mutant variants. Hemolytic activity was measured after 4 hours as the amount of released hemoglobin by photometric determination (A_{541nm}). **(B)** Binding of intact CyaA or its mutant variants to J774A.1 cells ($1x10^6$) was determined as the amount of total cell-associated AC enzyme activity upon incubation of cells with 1 µg/ml (5 nM) of the protein for 30 min at 4°C. cAMP intoxication was assessed by determining the intracellular concentration of cAMP generated in cells after 30 minutes of incubation of J774A.1 cells ($2x10^5$) with four different toxin concentrations from within the linear range of the dose-response curve (100, 50, 25 and 10 ng/ml). **(A, B)** Activities are expressed as percentages of intact CyaA activity and represent average values ± standard deviations from three independent determinations performed in duplicate with two different toxin preparations.

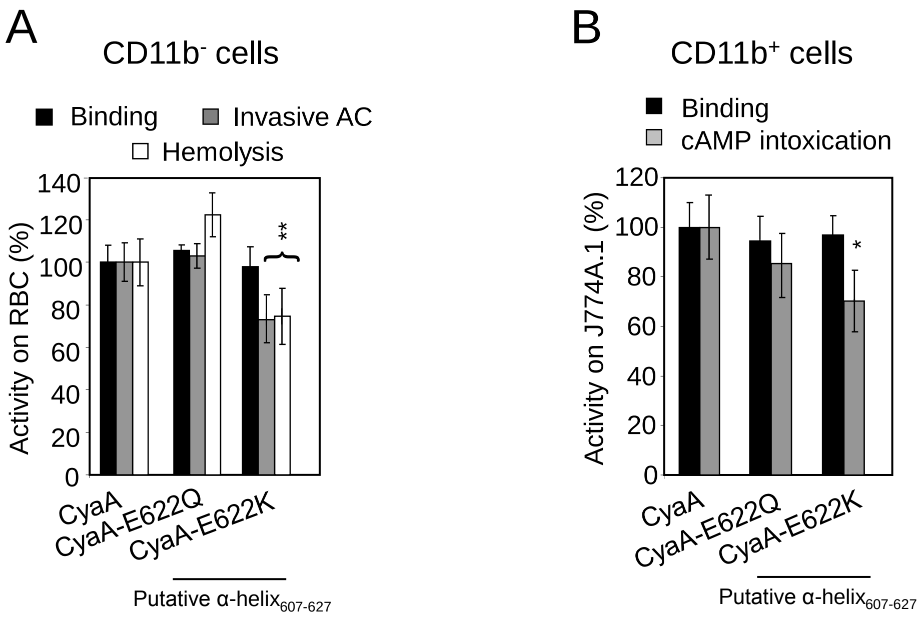

Supplementary Figure S3. Replacement of the negatively charged glutamate residue 622 by a neutral glutamine or by a oppositely charged lysine residue in the predicted α-helix (residues 607 to 627 of CyaA) has little or no impact on the toxin activities. (A, B) Preparations and analyses of the samples were performed as in the legend to Supplementary Fig. S2. Activities are expressed as percentages of intact CyaA activity and represent average values ± standard deviations from at least three independent determinations performed in duplicate with two different toxin preparations. Significant differences are indicated by asterisks (*, $p<0.05$; **, $p<0.01$).

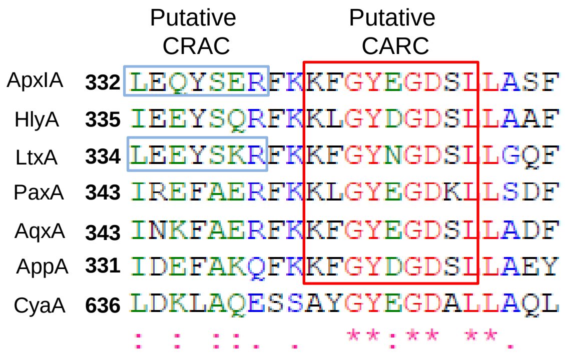

Supplementary Figure S4. Putative cholesterol binding motifs predicted in the hydrophobic domain of RTX toxins. ClustalW sequence alignment of a partial sequence of the acylated domain of CyaA and corresponding sequences of related RTX toxins. ApxIA, *Actinobacillus pleuropneumoniae* (uniprot P55128); HlyA, *Escherichia coli* (uniprot Q8G9Z4); LtxA, *Aggregatibacter actinomycetemcomitans* (uniprot P16462); PaxA, *Pasteurella aerogenes* (uniprot Q9RCG8); AqxA, *Actinobacillus equuli* (uniprot Q8KWZ9); AppA, *Kingella kingae* (uniprot F5S905); and CyaA, *Bordetella pertussis* (uniprot code P0DKX7). The putative conserved CARC (inverted CRAC) motifs are highlighted by a red frame. The predicted CRAC site of ApxIA located around the tyrosine residue at position 335 and the experimentally confirmed CRAC site of LtxA located around the tyrosine residue at position 337 [41] are highlighted by blue frames, * identity, : strongly similar, . weakly similar.
